# Regulation of cellular states via targeted phosphorylation of p53 using a nanobody-coupled kinase system

**DOI:** 10.1038/s41420-025-02821-1

**Published:** 2025-11-10

**Authors:** Hoe Eun Lim, Hae Yong Yoo

**Affiliations:** 1 Department of Health Sciences and Technology, Samsung Advanced Institute for Health Sciences and Technology, Sungkyunkwan University, Seoul, Republic of Korea; 2 Research Institute for Future Medicine, Samsung Medical Center, Seoul, Republic of Korea

**Keywords:** Phosphorylation, Targeted therapies, Checkpoint signalling

## Abstract

Phosphorylation participates in numerous signal transduction processes, including proliferation, differentiation, apoptosis, and cellular response to stimuli. Understanding its regulatory mechanisms is essential for advancing therapeutic interventions. In this study, we developed a target protein phosphorylation (TPP) system, consisting of a nanobody fused to a kinase domain, to investigate its ability to phosphorylate target proteins and regulate their cellular characteristics. We first verified that the nanobody-coupled kinase effectively phosphorylates GFP. Subsequently, we focused on p53 phosphorylation, identifying specific phosphorylation sites targeted by the system. This phosphorylation resulted in stabilization of p53 protein levels, inducing p21 expression, delaying cell cycle progression and suppressing cell growth. Furthermore, combining the TPP system with chemotherapeutic drugs (5-Fluorouracil and Oxaliplatin) enhanced cytotoxicity in colorectal cancer cells. The TPP system achieved p53 phosphorylation without external stimuli, inducing a DNA-damaged state in cells. In vivo, doxycycline-induced expression of the TPP system in a xenograft mouse model significantly inhibited tumor growth. This work demonstrates the ability to phosphorylate key regulatory proteins and alter cellular states, suggesting applications in studying phosphorylation-related pathways and developing therapies for diseases associated with dysregulated phosphorylation.

## Introduction

Phosphorylation is a fundamental biochemical process critical for the regulation of numerous cellular activities, such as cell proliferation, differentiation, and response to environmental stimuli [1]. It involves the covalent attachment of a phosphate group to proteins, primarily on serine, threonine, or tyrosine residues, mediated by kinases [2]. Through these modifications, phosphorylation can influence protein conformation, activity, stability, and interactions, playing a key role in signal transduction pathways [1, 2].

Among the proteins modulated by phosphorylation, the tumor suppressor protein p53 is particularly notable due to its pivotal role in maintaining genomic stability and preventing tumorigenesis [3]. p53 is activated in response to DNA damage and cellular stresses, leading to outcomes like cell cycle arrest, apoptosis, or senescence, depending on the cellular context [4]. The phosphorylation state of p53, especially at multiple serine residues, is crucial for its stability and functional activation [4, 5]. However, dysregulation of p53 phosphorylation is a common feature in many cancers, contributing to tumor progression and resistance to therapy [6].

Traditional approaches to study and manipulate phosphorylation, including broad-spectrum kinase inhibitors or activators, lack specificity and often cause off-target effects, complicating the interpretation of experimental results [7]. This lack of precision poses challenges, particularly in targeting specific p53 phosphorylation sites for therapeutic purposes [5]. To address these challenges, advancements in nanobody technology offer new avenues for precise targeting of proteins [8]. Nanobodies are small, single-domain antibodies derived from camelids known for their high specificity, stability, and ease of genetic modification [9, 10]. These characteristics make them suitable for engineering novel tools to study protein modifications and interactions.

In this study, we have innovatively developed the target protein phosphorylation (TPP) system to specifically phosphorylate target proteins such as p53. The TPP system consists of the fusion of a target-specific nanobody and a kinase domain. Our objective was to determine whether this targeted phosphorylation could effectively modulate p53 activity and alter cellular responses typically associated with DNA damage, without the need for external stimuli such as ionizing radiation. To this end, we investigated the effects of combining the TPP system with standard chemotherapeutic drugs in colorectal cancer cells and assessed its impact on tumor growth in a xenograft mouse model. This targeted approach allows for precise control over p53 phosphorylation, potentially leading to enhanced p53 stability, selective activation of specific pathways, and anti-tumorigenic effects. This approach not only provides insights into the precise regulation of p53 but also sets the stage for developing innovative therapeutic strategies to combat diseases driven by aberrant phosphorylation.

## Results

### Development of nanobody-coupled kinase system for target protein phosphorylation

**Fig. 1:**
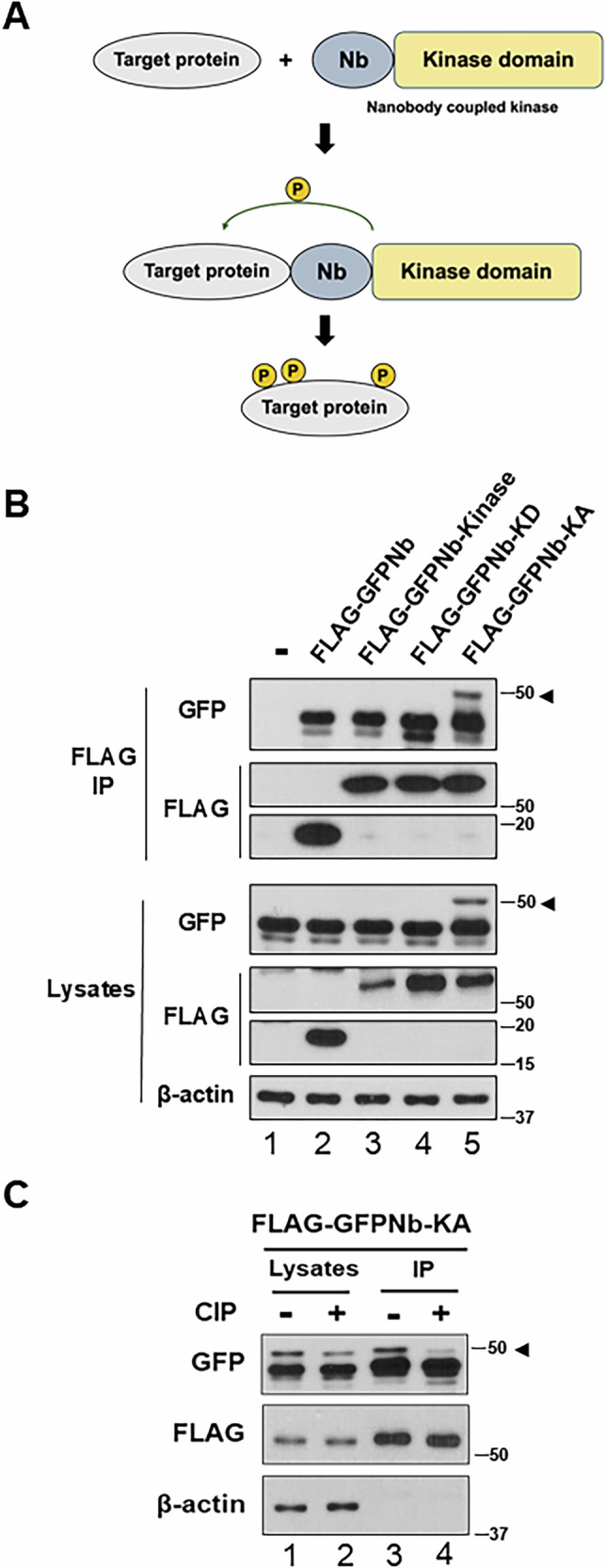
Establishment of target protein phosphorylation using nanobody-coupled kinase; GFP specific nanobody-coupled kinase phosphorylates GFP. **A** Schematic representation of target protein phosphorylation (TPP) utilizing the nanobody-coupled kinase. Nb (Nanobody), variable domain of the heavy chain of heavy-chain antibodies. **B** Stable GFP-expressing HEK 293 T cells were transfected with various constructs: untransfected control (lane 1), a vector expressing FLAG-GFP nanobody (lane 2), FLAG-GFP nanobody-fused wild-type kinase (lane 3), FLAG-GFP nanobody-fused kinase-dead (FLAG-GFPNb-KD) (lane 4), and FLAG-GFP nanobody-fused kinase-active (FLAG-GFPNb-KA) (lane 5). FLAG-tagged nanobody kinases were immunoprecipitated after 48 h, followed by immunoblotting with anti-GFP and anti-FLAG antibodies. Lysates were also immunoblotted. Arrowheads indicate phosphorylated GFP. **C** To determine if the shifted band of GFP could be eliminated, cell lysates and immunoprecipitates were treated with calf intestinal phosphatase (CIP) for 1.5 h at 37 °C. Following CIP treatment, lysates and immunoprecipitates (IP) obtained using FLAG antibodies were immunoblotted with anti-GFP, anti-FLAG, and anti-β-actin antibodies. Numbers on the right indicate molecular weight markers (kDa).

To achieve target protein phosphorylation, we developed a nanobody-coupled kinase, which involves the fusion of a nanobody and a kinase domain. Through the evaluation of various kinase domains, we identified that the PLK1 kinase domain functions effectively. Figure 1A and Supplementary Fig. S1 show a schematic of the experimental design, detailing the different constructs used: a nanobody fused to a wild-type PLK1 kinase domain (Nb-Kinase), a nanobody fused to a kinase-active mutant (Nb-KA), and a nanobody fused to a kinase-dead mutant (Nb-KD) [11–13]. Using a nanobody-coupled kinase consisting of a GFP-specific nanobody and the PLK1 kinase domain, we confirmed the phosphorylation of GFP in cell lines expressing GFP [14, 15].

Following transfection with these various constructs, as depicted in Fig. 1B, we observed a significant increase in phosphorylated GFP (indicated by the arrowhead) only in cells transfected with the Nb-KA construct (lane 5). Phosphorylation was not observed in cells transfected with untransfected control (lane 1), the GFP nanobody alone (lane 2), the wild-type Nb-Kinase (lane 3) or the Nb-KD construct (lane 4). This indicated that the kinase activity, specifically within the context of the Nb-KA, is responsible for GFP phosphorylation. Immunoblotting with anti-FLAG antibody confirmed successful expression of the FLAG-tagged nanobody-kinase constructs. To confirm the specificity of the observed GFP phosphorylation, we treated the lysates and immunoprecipitates with calf intestinal phosphatase (CIP), as shown in Fig. 1C. Treatment with CIP eliminated the upper, slower-migrating phosphorylated GFP band, demonstrating that the observed shift in the GFP band was indeed due to phosphorylation. This observation further strengthens the finding that the nanobody-fused kinase system is directly responsible for the phosphorylation of the target protein GFP. These results demonstrate that the kinase activity, specifically within the context of the nanobody-kinase fusion, is responsible for the observed GFP phosphorylation.

### Nanobody-coupled kinase system enhances p53 phosphorylation and stabilization

**Fig. 2:**
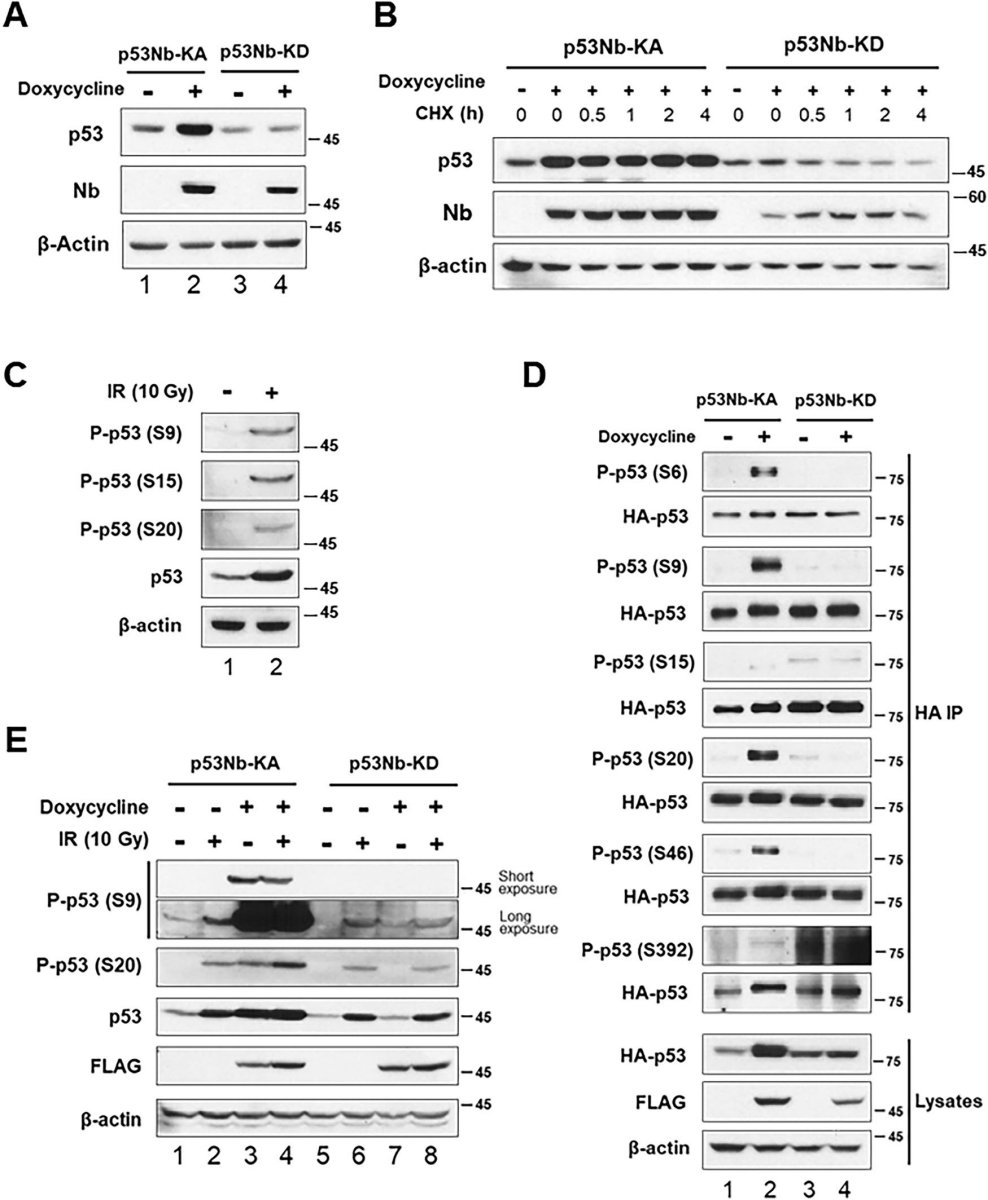
Analysis of p53 phosphorylation by nanobody-kinase system and ionizing radiation (IR). **A** Western blot analysis of p53 levels in HCT116 cells expressing doxycycline-inducible a p53-nanobody-kinase-active (p53Nb-KA) or a p53-nanobody-kinase-dead (p53Nb-KD). Cells were treated with or without doxycycline for 48 h and lysates were subjected to immunoblotting using indicated antibodies. Nb antibody confirmed nanobody-kinase expression. β-actin served as a loading control. **B** p53 protein stability was assessed by immunoblotting following cycloheximide (CHX, 10 µM) treatment of cells expressing p53Nb-KA or p53Nb-KD, with or without 20 h doxycycline pre-treatment. Cells were harvested at the indicated times. Nb antibody confirmed p53-nanobody-kinase expression; β-actin served as a loading control. **C** Immunoblotting of HCT116 cell lysates with phospho-specific p53 antibodies (Serine 9 (S9), Serine 15 (S15), Serine 20 (S20)), total p53, and β-actin (loading control) was performed to assess p53 phosphorylation 2 h after 10 Gy of ionizing radiation (IR) treatment. **D** To compare p53 phosphorylation induced by the nanobody-kinase system, HCT116 cells constitutively expressing HA-p53-GFP and doxycycline-inducible p53Nb-KA or p53Nb-KD were treated with doxycycline for 48 h. Immunoblot analysis examined p53 phosphorylation at S6, S9, S15, S20, S46, and S392 in cells with or without doxycycline induction. To correct for a significantly higher p53 level observed in lane 2 of the lysate blot (Fig. 2A), p53 levels were normalized following HA immunoprecipitation (IP) to ensure accurate comparison of phosphorylation across samples. Cell lysates were immunoblotted with anti-p53, anti-FLAG, and anti-β-actin antibodies. **E** Western blot analysis of p53 phosphorylation at S9 and S20, total p53 levels, FLAG-tagged nanobody-kinase expression, and β-actin (loading control) in HCT116 stable cells expressing either p53Nb-KA or p53Nb-KD constructs. Cells were treated with or without doxycycline for 48 h and exposed to 10 Gy of IR. Cell lysates were prepared 2 h after 10 Gy IR. Short and long exposure images are shown for phospho-p53 (S9).

p53 plays a pivotal role among proteins regulated by phosphorylation, governing key cellular processes such as DNA damage response and tumor suppression [3, 5]. Thus, we chose p53 to assess our TPP system’s capability to induce phosphorylation. We utilized a doxycycline-inducible system expressing either a p53 nanobody kinase-active (p53Nb-KA) or a kinase-dead (p53Nb-KD) fusion protein. The nanobody component of these fusion proteins was based on a previously published p53-specific nanobody [16]. Doxycycline treatment induced expression of both p53Nb-KA and p53Nb-KD constructs, as confirmed by immunoblotting with a Nb antibody (Fig. 2A). A marked increase in total p53 protein levels was observed in doxycycline-induced p53Nb-KA cells (Fig. 2A, lane 2) but not in cells expressing the kinase-dead p53Nb-KD construct or under other conditions, including expression of only the p53 nanobody or the kinase domain (Supplementary Fig. S2). To further investigate the impact of the nanobody-kinase system on p53 protein levels, we examined p53 stability. Cells expressing p53Nb-KA or p53Nb-KD, with or without doxycycline induction, were treated with cycloheximide (CHX) to inhibit new protein synthesis. This allowed us to monitor the half-life of existing p53 protein. p53 protein levels were subsequently analyzed by immunoblotting (Fig. 2B). In cells expressing the active p53Nb-KA construct, p53 protein levels were significantly higher at all time points compared to the control, suggesting that this system stabilizes the p53 protein. In contrast, p53 protein levels decreased more rapidly in cells expressing the p53Nb-KD construct. These data further demonstrate that activation of the nanobody-kinase system enhances p53 stability. This is consistent with the established role of phosphorylation in regulating p53 stability [4, 17]. Importantly, Supplementary Fig. S2 demonstrates that this effect requires the presence of both the p53 nanobody and an active kinase domain.

Next, we assessed p53 phosphorylation at various known sites (S9, S15, S20) in cells exposed to 10 Gy of ionizing radiation (IR) (Fig. 2C). As expected, IR treatment resulted in increased phosphorylation at these sites [4]. Importantly, we also observed a concomitant increase in total p53 protein levels upon IR treatment, consistent with the known stabilizing effect of p53 phosphorylation in response to cellular stress [18–20]. This served as a positive control, validating the experimental setup and confirming the responsiveness of p53 to stress-inducing stimuli. The significant increase in p53 levels observed in cells expressing p53Nb-KA (Fig. 2A, lane 2) and the lack of increase in cells expressing p53Nb-KD strongly suggest that this is a direct consequence of the p53Nb-KA-mediated p53 phosphorylation.

To directly compare the effects of our nanobody-kinase system with IR-induced p53 phosphorylation, we performed a more comprehensive analysis, examining multiple phosphorylation sites (S6, S9, S15, S20, S46, S392) in cells expressing either p53Nb-KA or p53Nb-KD constructs (Fig. 2D). To facilitate p53 enrichment for phosphorylation analysis, we generated a cell line constitutively expressing HA-GFP-p53. Following doxycycline induction in p53Nb-KA and p53Nb-KD expressing cells, HA immunoprecipitation (IP) was performed to isolate the p53 protein. However, as shown in Fig. 2A (lane 2), doxycycline induction in p53Nb-KA cells resulted in significantly higher total p53 protein levels compared to other conditions. To ensure accurate comparison of p53 phosphorylation levels across all samples, we normalized p53 levels following IP. Specifically, after an initial Western blot, band intensities for total p53 were quantified by densitometry, and protein loading for subsequent Western blots was adjusted to achieve equivalent total p53 levels in each sample. The results revealed that the expression of the active p53Nb-KA construct, upon doxycycline induction, led to increased p53 phosphorylation at several sites (S6, S9, S20, S46), whereas the kinase-dead p53Nb-KD construct did not induce significant phosphorylation. Importantly, no significant phosphorylation was observed at S15 or S392 under any of the conditions tested. This indicates a selective effect of the nanobody-kinase system on specific p53 phosphorylation sites and highlights a direct link between the kinase activity of the nanobody-fusion protein and p53 phosphorylation at these particular sites.

Finally, we investigated the effects of both doxycycline induction and IR treatment on p53 phosphorylation (Fig. 2E), focusing on S9 and S20. We observed increased phosphorylation at both sites in cells expressing p53Nb-KA and treated with doxycycline, with or without additional IR treatment. The long exposure of the S9 blot (Fig. 2E) reveals significantly higher levels of phosphorylation at S9 in cells expressing the active nanobody-kinase construct compared to those treated with IR alone, indicating a substantially more potent effect of the nanobody-kinase system on p53 phosphorylation at this site.

In summary, our results demonstrate that the nanobody-kinase system is capable of inducing p53 phosphorylation at multiple sites (S6, S9, S20, S46), mimicking some, but not all, of the effects observed following exposure to ionizing radiation, including the increase in p53 protein levels. Importantly, the system did not induce phosphorylation at S15 or S392. Furthermore, the system appears to increase p53 protein levels, likely due to enhanced p53 stabilization. The phosphorylation induced by the nanobody-kinase system is dependent upon an active kinase domain, and, at least at S9, is demonstrably more potent than that induced by IR treatment alone.

### Downstream effects of nanobody-kinase mediated p53 phosphorylation: selective gene activation

**Fig. 3:**
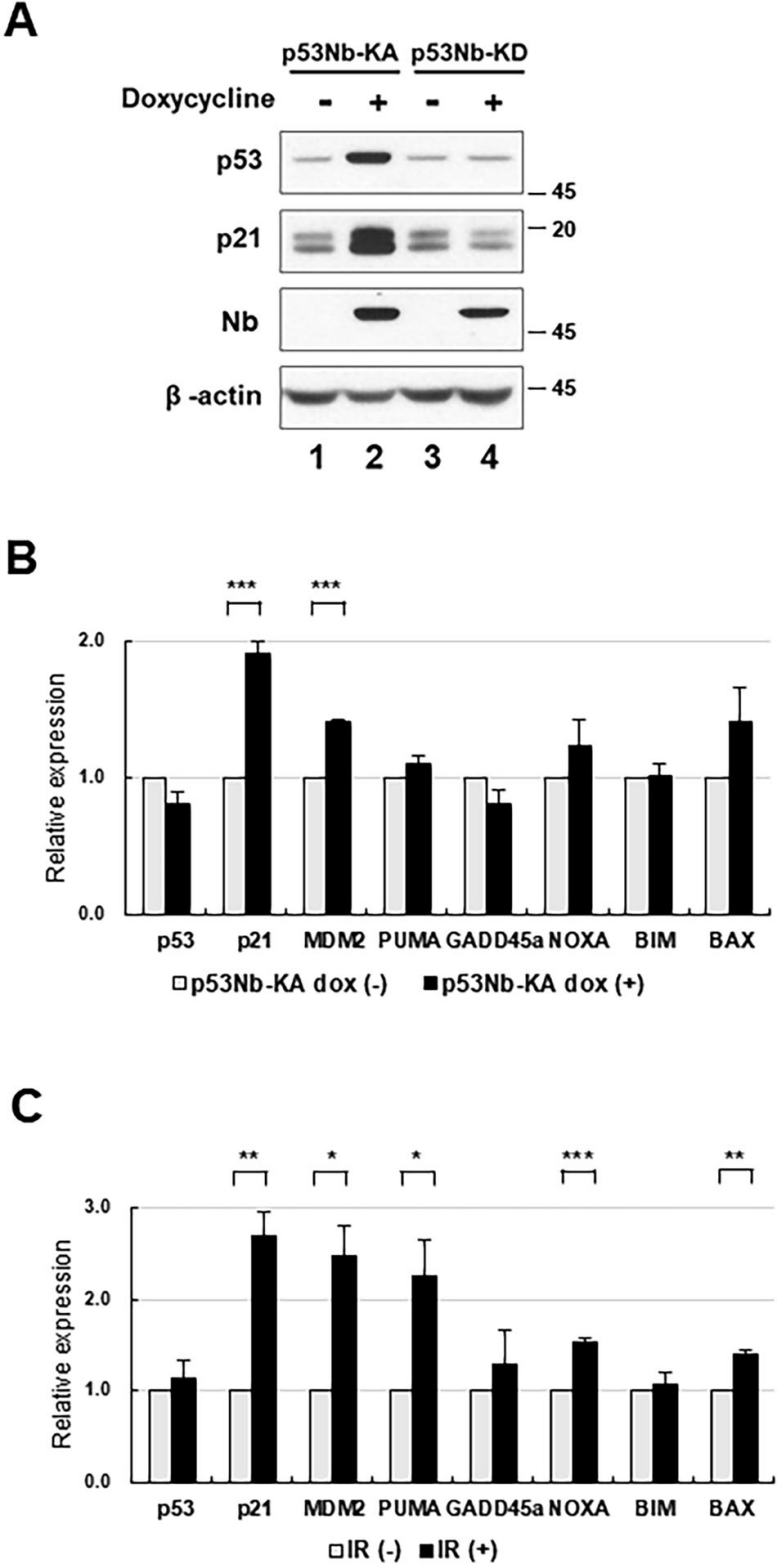
Effects of p53 phosphorylation on p53 target gene expression and protein stability. **A** Immunoblot analysis of p53, p21, and β-actin protein levels in HCT116 stable cells expressing either p53Nb-KA or p53Nb-KD with or without doxycycline induction. Cells were induced with doxycycline for 48 h and cell lysates were prepared. The Nb blot shows the expression levels of the nanobody-kinase fusion proteins. β-actin serves as a loading control. **B** Quantitative analysis of p53, p21, MDM2, PUMA, GADD45a, NOXA, BIM, and BAX mRNA expression levels in HCT116 cells expressing p53Nb-KA with or without doxycycline induction. **C** Expression levels following IR treatment. Cells were harvested 2 h after exposure to 10 Gy of IR. Relative expression levels are normalized to the control (defined as p53Nb-KA without doxycycline induction for panel B and non-irradiated control cells for panel **C**). Data are presented as mean ± SEM (*n* = 3; *p < 0.05, **p < 0.01 and ***p < 0.001).

To investigate the downstream effects of p53 phosphorylation mediated by our nanobody-kinase system, we examined the expression of known p53 target genes. Figure 3A shows the protein levels of p53 and its downstream target p21 in cells expressing either the p53Nb-KA or p53Nb-KD construct, with or without doxycycline induction [21, 22]. The expression of the nanobody-kinase fusion proteins was confirmed by immunoblotting with a Nb antibody. Doxycycline induction led to increased levels of p53 in cells expressing the p53Nb-KA construct (Fig. 3A, lane 2). Consistently, p21 protein levels were significantly elevated in the doxycycline-induced p53Nb-KA cells (lane 2) but the p53Nb-KD construct did not induce a significant increase in p21 expression (lane 4). These results indicate that the kinase activity of the nanobody fusion stabilized p53, which in turn activated the transcriptional pathway, leading to increase in p21 levels [21, 22].

To further explore the transcriptional effects of p53Nb-KA, we performed a quantitative PCR (qPCR) analysis of p53 target gene expression, including p21, MDM2, PUMA, GADD45a, NOXA, BIM, and BAX [21–28]. The results in Fig. 3B show that induction of the p53Nb-KA construct with doxycycline significantly increased the expression of p21 and MDM2, but PUMA, GADD45a, NOXA, BIM, and BAX were not significantly altered. This further supports the conclusion that the nanobody-kinase system selectively activates a subset of p53 target genes, primarily those involved in cell cycle arrest. To compare this to a canonical DNA damage response, we additionally analyzed gene expression following IR treatment. In contrast to the selective activation by the nanobody-kinase system, IR treatment strongly induced not only p21 and MDM2, but also pro-apoptotic targets such as PUMA, NOXA, and BAX (Fig. 3C). These findings demonstrate that while IR broadly engages the p53 transcriptional activation, our nanobody-kinase system preferentially drives a selective activation of p21and MDM2. In summary, the activation of the p53 nanobody-kinase system leads to selective activation of specific p53 downstream target genes, suggesting the potential of this approach to manipulate p53-mediated cellular functions [29].

### p53 phosphorylation inhibits cell growth and invasion

**Fig. 4:**
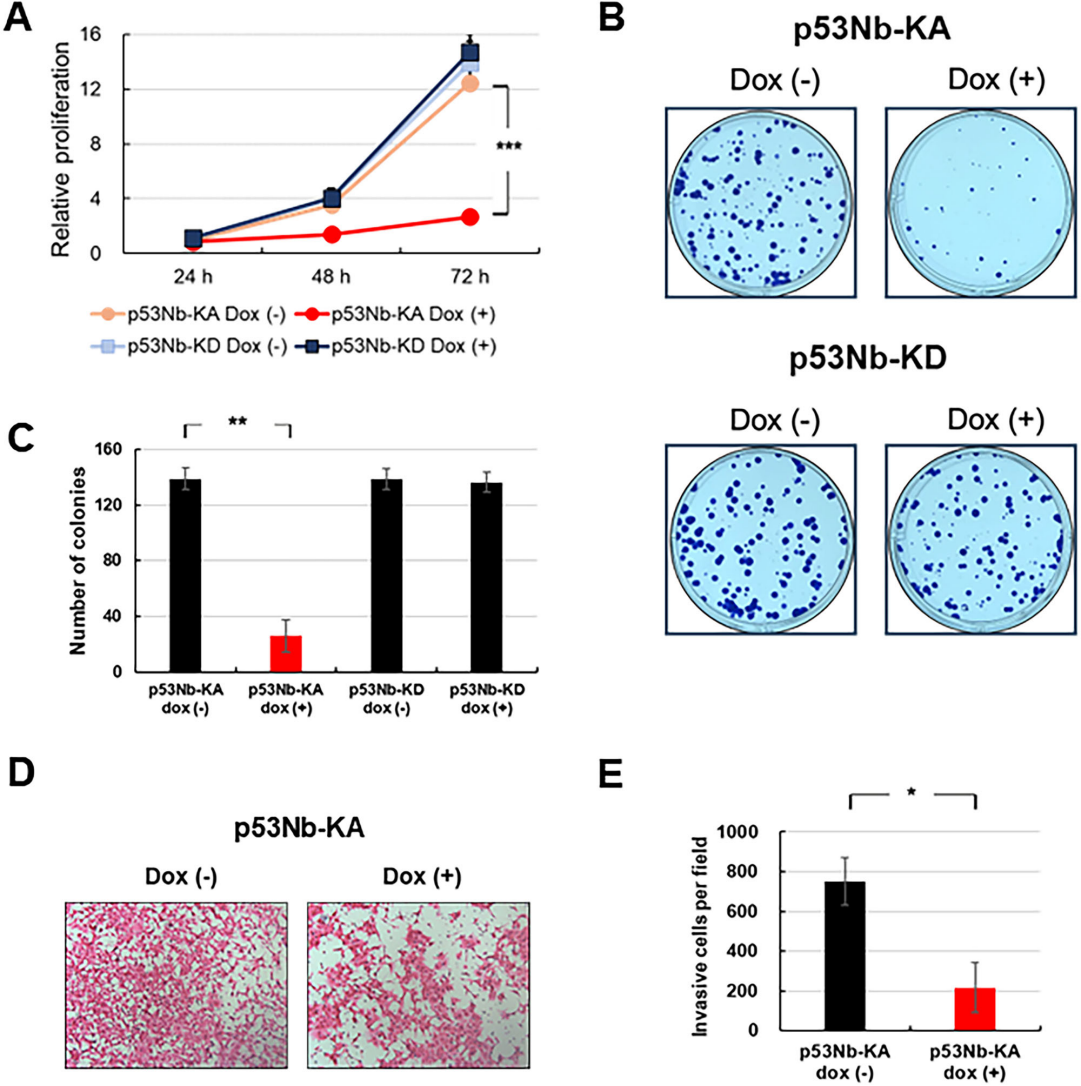
Effects of p53 phosphorylation on cell proliferation, colony formation, and invasion. **A** Cell proliferation rates (24–72 h) were determined for HCT116 cells expressing p53Nb-KA or p53Nb-KD, with or without doxycycline induction, using the Cell Counting Kit-8 (CCK-8) assay. Data represent the mean ± SEM (*n* = 3; ***p < 0.001). **B** Representative images of colony formation assays performed on HCT116 stable cells expressing p53Nb-KA or p53Nb-KD. Cells were cultured for 10 days, with doxycycline added twice per week to induce expression of the nanobody-kinase constructs. **C** Quantification of colony numbers from colony formation assays shown in (**B**). Data represent the mean ± SEM (*n* = 3; **p < 0.01). **D** Representative images of invasion assays performed on cells expressing p53Nb-KA with or without doxycycline induction. Invasion ability was measured after incubation for 72 h. **E** Quantification of invasive cells per field of view from invasion assays shown in (**D**). Data represent the mean ± SEM (*n* = 3; *p < 0.05).

p53 is a well-known tumor suppressor, and its stabilization can regulate various cellular functions [6]. Thus, we examined whether p53 stabilization by our TPP system influences these cellular functions. We determined the effects of p53 phosphorylation on cell growth and invasiveness. First, we assessed cell proliferation using CCK-8 assay, a widely used method to quantify viable cells. Figure 4A shows that doxycycline induction of the p53Nb-KA construct significantly reduced cell proliferation compared to the uninduced p53Nb-KA cells. This growth suppression was not observed in the p53Nb-KD cells (Fig. 4A), suggesting that p53 phosphorylation is responsible for the observed anti-proliferative effect [21, 30]. Next, we investigated the effects on colony formation (Fig. 4B, C). Consistent with the cell proliferation results, doxycycline induction of p53Nb-KA led to a significant decrease in the number of colonies formed. In contrast, the p53Nb-KD construct showed no significant change in colony formation upon doxycycline induction. These findings further support a role for p53 phosphorylation in inhibiting cell growth.

Finally, we evaluated the effects on cell invasion, an important characteristic of metastasis (Fig. 4D, E) [31]. Representative images of invasion assays are shown in Fig. 4D. Quantitative analysis revealed a significant reduction in the number of invasive cells in the doxycycline-induced p53Nb-KA cells. In summary, the results presented in Fig. 4 demonstrate that the activation of the p53-nanobody-kinase system significantly suppresses cell proliferation, colony formation, and invasiveness. These findings suggest that this system has the potential to be employed as a tool for cancer therapeutics targeting p53 pathway activation [32]. These observations support the hypothesis that specific p53 phosphorylation, mediated by our system, exerts significant anti-tumorigenic effects.

### p53 phosphorylation delays cell cycle progression

**Fig. 5:**
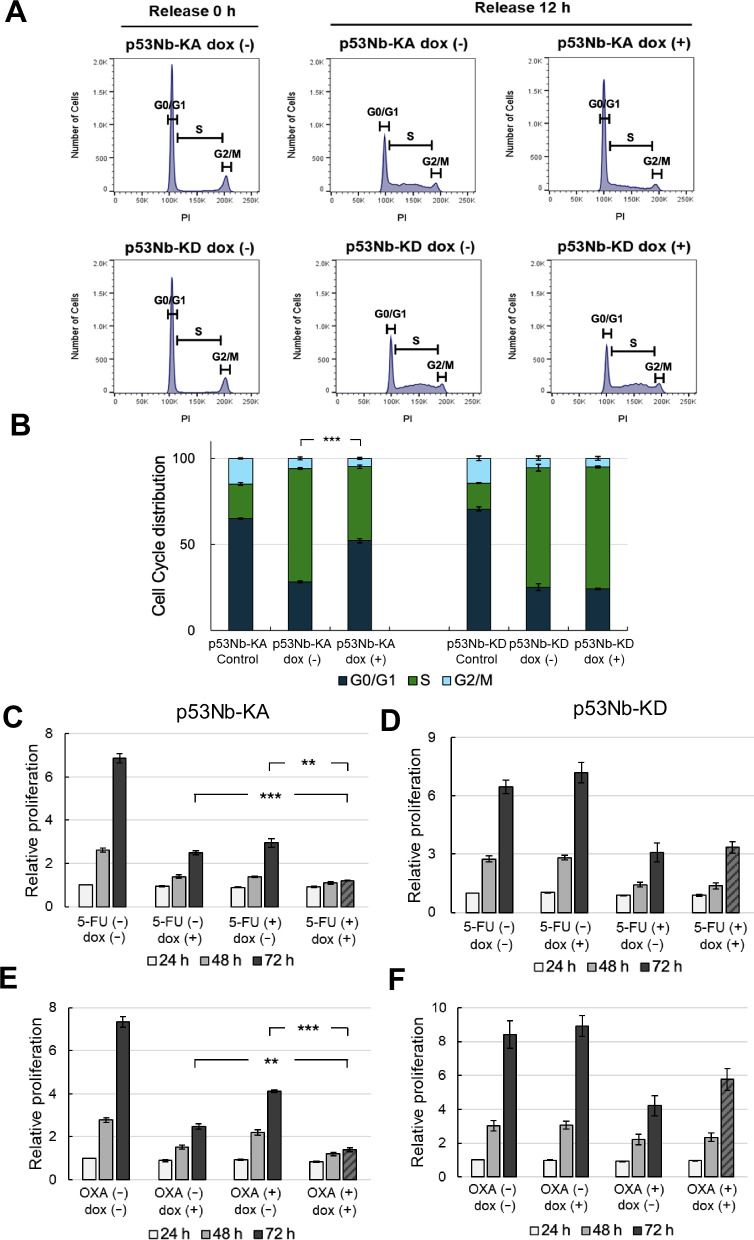
Effects of p53 phosphorylation on cell cycle progression and cancer chemotherapeutic drugs. **A** Flow cytometry analysis of HCT116 cells expressing p53Nb-KA or p53Nb-KD determined cell cycle distribution (G0/G1, S, G2). Cells were synchronized by serum starvation for 24 h, with or without doxycycline treatment during the final 8 h of starvation. After release into serum-containing medium in the presence or absence of doxycycline, cells were harvested 12 h post-release and analyzed for cell cycle distribution using propidium iodide (PI) staining. **B** Quantification of cell cycle distribution from flow cytometry analysis shown in (**A**). Error bars represent the mean ± SEM (*n* = 3). Statistical significance was determined for the G0/G1 phase population (***p < 0.001). **C–F** HCT116 stable cells expressing p53Nb-KA or p53Nb-KD were treated with either 2 μM 5-Fluorouracil (5-FU) (**C, D**) or 2 μM Oxaliplatin (OXA) (**E, F**), with or without doxycycline induction. Cell proliferation was measured using the CCK-8 assay at 24, 48, and 72 h. Data are shown as mean ± SEM (*n* = 3); significance levels are indicated as ***p < 0.001, and **p < 0.01.

To investigate the impact of sustained p53 activation on cell cycle progression, we analyzed cell cycle distribution following serum stimulation after serum starvation. Cells were serum-starved for 24 h in serum-free medium for synchronization [33]. Doxycycline was added 8 h before the end of the starvation period to induce p53Nb-KA or p53Nb-KD expression. Complete medium with or without doxycycline, was then added to progress into cell cycle. Cells were harvested for flow cytometry analysis 12 h after release from serum starvation. Figure 5A shows the cell cycle distribution profiles for control cells and cells expressing either p53Nb-KA or p53Nb-KD constructs, with or without doxycycline treatment during the 12-h serum stimulation period. As seen in Fig. 5A, serum-starvation caused cell cycle arrest, primarily in the G0/G1 phase, in both the control p53Nb-KA and the control p53Nb-KD expressing cells. However, following a 12-h release from starvation, the p53Nb-KD cells progressed through the cell cycle and efficiently entered the S phase. In contrast, cells expressing the active p53Nb-KA construct showed a significantly different distribution, with a greater percentage of cells remaining in the G0/G1 phase and fewer cells in the S and G2 phases compared to cells expressing p53Nb-KD. This is consistent with the known role of p53 in cell cycle arrest at the G1 checkpoint [34, 35]. In contrast, cells expressing p53Nb-KD showed no significant change in cell cycle distribution upon induction with doxycycline, which supports the hypothesis that p53 phosphorylation is essential for cell cycle regulation. Figure 5B presents a quantification of the cell cycle data, highlighting the significant differences in cell cycle distribution, particularly in the G1 phase, among doxycycline-treated p53Nb-KA cells. In summary, these results demonstrate that sustained activation of the p53 pathway via the p53Nb-KA leads to a prolonged G0/G1 cell cycle arrest. This observation further supports the potent influence of this system on cell cycle regulation.

### p53 phosphorylation enhances drug cytotoxicity in colorectal cancer cells

To investigate whether p53 phosphorylation enhances the effect of chemotherapeutic drugs, we performed cell proliferation assays using doxycycline-inducible HCT116 stable cells expressing p53Nb-KA or p53Nb-KD. Cells were treated with or without doxycycline in combination with either 5-FU [36] or Oxaliplatin [37] and evaluated cell growth at 24, 48, and 72 h. p53Nb-KA cells treated with the combination of doxycycline and either 5-FU or Oxaliplatin exhibited significant decreases in cell proliferation compared to either treatment alone, suggesting a synergistic effect where activating p53 through the nanobody-kinase enhances the cytotoxic effect of these chemotherapeutic drugs (Fig. 5C and E). In contrast, p53Nb-KD cells treated with the combination of doxycycline and either 5-FU or Oxaliplatin showed a less pronounced decrease in cell proliferation compared to the chemotherapeutic drug alone (Fig. 5D and F). These data suggest that activating p53 through the nanobody-kinase system (p53Nb-KA) enhances the effectiveness of both 5-FU and Oxaliplatin in HCT116 colorectal cancer cells, highlighting the potential of combining targeted p53 activation with traditional chemotherapeutic drugs to improve treatment outcomes. The lack of enhancement with kinase-dead construct (p53Nb-KD) indicates that the kinase activity of the nanobody-kinase is crucial for the observed synergistic effects.

### p53 phosphorylation in a xenograft mouse model significantly inhibits tumor growth

**Fig. 6:**
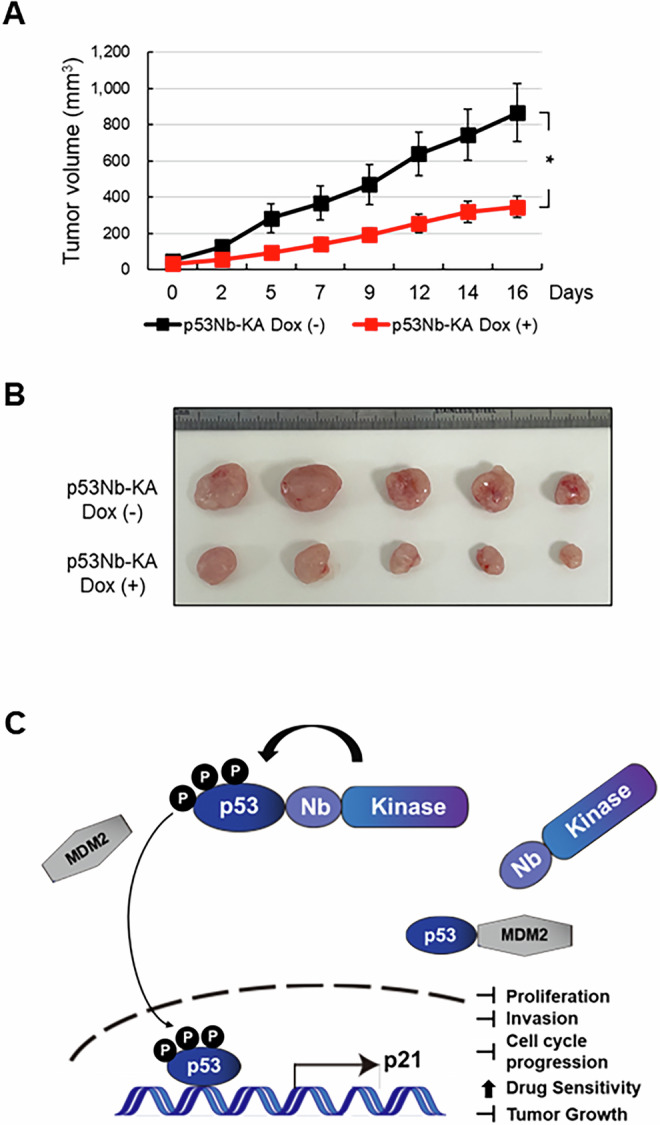
Inhibition of tumor growth in vivo by targeted p53 phosphorylation. **A** Doxycycline-inducible HCT116 stable cells expressing p53Nb-KA were subcutaneously injected into the left flank of BALB/c-nude mice (*n* = 5 per group). Mice were fed with a diet containing doxycycline for one group until the end of the experiment. Tumor volumes were measured using a digital caliper three times a week for 16 days. Data are presented as mean ± SEM; *p < 0.05. **B** Images of extracted tumors from the p53Nb-KA group with or without doxycycline induction at the experimental end point. **C** Schematic model illustrating the proposed mechanism of action of the p53-nanobody-kinase system. Doxycycline-induced expression of the p53Nb-KA construct leads to p53 phosphorylation, resulting in p53 stabilization, increased p21 expression, and subsequent suppression of cell proliferation and invasion, induction of cell cycle arrest, enhanced drug sensitivity, and inhibition of tumor growth.

Finally, we examined the impact of p53 phosphorylation on tumor growth in a mouse xenograft model using the doxycycline inducible HCT116 stable cells expressing p53Nb-KA. Targeted p53 phosphorylation, induced by doxycycline-containing diet significantly inhibited tumor growth in vivo (Fig. 6A). The smaller tumor volumes in the doxycycline-treated group indicated that activating p53 phosphorylation has a significant anti-tumor effect. Visual confirmation of the reduced tumor size in the doxycycline-treated group was provided by image of the extracted tumors (Fig. 6B). These results suggest that the nanobody-kinase system targeting p53 phosphorylation is a viable strategy for inhibiting tumor growth in colorectal cancer.

These findings are summarized in a proposed model (Fig. 6C). This study demonstrates the successful development and application of a novel nanobody-kinase system for targeted p53 phosphorylation. Activation of this system resulted in enhanced p53 stability and selective phosphorylation at specific sites. Downstream consequences included increased p21 expression, cell cycle arrest at the G1 phase, and significant suppression of cell proliferation, colony formation, and invasiveness in vitro. Furthermore, combination drug treatment with 5-FU and Oxaliplatin in HCT116 stable cells expressing p53Nb variants revealed enhanced inhibition of cell proliferation in the presence of doxycycline, indicating the therapeutic potential of the system in sensitizing tumor cells to chemotherapeutic agents. In vivo, doxycycline-induced expression of p53Nb-KA in a mouse model led to significant tumor growth suppression, further underscoring the potential of this technology for precise manipulation of p53 signaling and its utility as a tool for cancer therapeutics.

## Discussion

The findings of this study highlight the significant potential of using a nanobody-coupled kinase system to specifically phosphorylate target proteins such as p53, thereby influencing cellular behavior and signaling pathways. This approach offers several advantages over traditional methods of studying protein phosphorylation, which often rely on broad-spectrum kinase inhibitors and can lead to off-target effects that complicate results [7].

Our results demonstrate that the nanobody-coupled kinase system successfully phosphorylates p53 at specific sites, namely Ser6, Ser9, Ser20, and Ser46, leading to changes in p53 stability and activity similar to those induced by ionizing radiation [3, 4]. The phosphorylation of p53 resulted in increased p21 and MDM2 expression and delayed cell cycle progression, mirroring typical responses to DNA damage [5, 21]. These findings underscore the system’s ability to mimic stress responses without external stimuli, providing a controlled and precise method to study phosphorylation-dependent signaling. p53 regulates a wide array of target genes, resulting in diverse cellular outcomes including cell cycle arrest, DNA repair, senescence, and apoptosis, depending on the specific cellular context [4]. However, in our system, p53 activation preferentially induced cell cycle arrest, as evidenced by the significant upregulation of the cell cycle arrest-associated gene p21, while the expression of the pro-apoptotic gene PUMA remained unchanged [21, 24, 25].

The observed G1 cell cycle arrest, coupled with the significant reduction in cell proliferation, colony formation, and invasiveness, strongly indicates that the nanobody-kinase system exerts significant anti-tumorigenic effects. These results are consistent with previous studies demonstrating the critical role of p53 in regulating cell cycle progression and suppressing tumor growth [21, 32]. The significant reduction in invasiveness is particularly relevant, suggesting a potential to reduce metastatic spread, a major challenge in cancer treatment. [31, 38].

The enhanced potency of the nanobody-kinase system at S9 of p53 compared to IR-induced phosphorylation is noteworthy and may relate to factors such as the sustained and localized nature of nanobody-mediated kinase activity. Further investigation is warranted to elucidate the mechanisms underlying this enhanced potency and to explore potential synergistic effects with other therapeutic approaches [39]. While the capability to phosphorylate target proteins has been established, one of the significant challenges that the nanobody-coupled kinase system faces is achieving precise phosphorylation at exact sites within target proteins. Addressing this hurdle of specificity in site-directed phosphorylation is crucial for maximizing the potential of this technology.

The stability and specificity of nanobodies [9] enable targeted manipulation of phosphorylation events with minimal off-target activity. This specificity is particularly important for therapeutic applications, where precise modulation of signaling pathways can significantly impact treatment efficacy and safety [40]. The precise targeting and modulation of phosphorylation, dysregulated in many diseases including cancer [6, 41, 42], offers potential for novel therapeutic strategies.

Moreover, the application of such a system could extend beyond cancer research. It might be applicable in understanding signaling pathways in other diseases where phosphorylation plays a central role, such as neurodegenerative disorders and metabolic diseases [41, 42]. The modularity of nanobody-coupled systems could allow researchers to redesign and adapt them for various cellular contexts and protein targets, underscoring their potential versatility in biomedical research. Future investigations could explore solutions to enhance site-specificity, as well as the scalability of this system and its potential in in vivo studies to assess its efficacy and safety in more complex biological environments. Additionally, expanding this approach to other proteins and pathways could offer deeper insights into the intricate network of cellular signaling and its implications for health and disease.

In conclusion, the nanobody-kinase system represents a promising new tool for precisely manipulating p53 signaling. Its ability to induce targeted phosphorylation, enhance p53 stability, selectively activate downstream effectors, and suppress tumorigenic properties suggests significant therapeutic potential. Our findings, demonstrating synergistic enhancement of chemotherapeutic drug cytotoxicity in colorectal cancer cells and significant inhibition of tumor growth in a xenograft mouse model, provide a strong rationale for further investigation of this system as a cancer therapy. While further studies are needed to evaluate its efficacy, safety, and potential in combination therapies, the results presented here offer compelling evidence for its promise as a novel therapeutic strategy for cancers aimed at restoring and strengthening p53 activity.

## Materials and methods

### Plasmid construction

FLAG-tagged GFPNb-Kinase and p53Nb-Kinase constructs were generated by PCR amplification of each fragment. The resulting PCR fragments were then cloned downstream of a doxycycline-inducible promoter in a pLVX vector. The sequences for the GFP nanobody (GFPNb) [14, 15] and p53 nanobody (p53Nb) [16] were derived from previously published sequences and the PDB Bank. Constitutively active kinase and kinase dead mutants were generated using site-directed mutagenesis. For immunoprecipitation experiments, an HA-GFP-p53 fusion construct was generated by PCR amplification and cloned downstream of the CMV promoter in pCDH vector. Detailed sequence information is provided in Supplementary Table S1.

### Cell culture and cell lines

HEK 293 T and HCT116 cells were cultured in DMEM and RPMI1640 medium (Welgene, Cat# LM 001-05, LM 011-01), respectively, supplemented with 10% fetal bovine serum (FBS; Welgene, Cat# S101-01), 100 U/mL penicillin and 100 µg/mL streptomycin (Gibco, Cat# 15140122) at 37°C with 5% CO_2_. Cells were routinely tested for mycoplasma contamination and authenticated by short tandem repeat (STR) profiling.

### Transfection and stable cell lines

HEK 293 T cells were transiently transfected with FLAG-tagged GFPNb-KA or GFPNb-KD expression plasmids using Lipofectamine 3000 (ThermoFisher Scientific, Cat# L3000008). Stable HCT116 cell lines expressing doxycycline-inducible FLAG-tagged p53Nb-KA or p53Nb-KD were established by hygromycin selection (200 µg/mL) of resistant clones 48 h post-transfection. Nanobody coupled kinase expression was induced with 1 µg/mL doxycycline (Sigma-Aldrich, Cat# D9891).

### Immunoprecipitation and immunoblotting

HCT116 stable cells expressing nanobody-kinase fusions were treated with 1 µg/ml doxycycline. For irradiation experiment, cells were harvested 2 h following exposure to 10 Gy of ionizing radiation. Cells were lysed with lysis buffer (50 mM Tris, 150 mM NaCl, 1 mM EDTA, 0.5% NP-40, 1% Triton X-100) containing protease inhibitor and phosphatase inhibitor. Cell lysates were prepared by incubating on ice for 30 min, followed by centrifugation (13,000 rpm, 4°C). Supernatants were incubated overnight at 4°C with HA antibody (Santa Cruz Biotechnology, Cat# sc-7392), then with Dynabeads protein G (Invitrogen, Cat# 10004D) for 3 h at 4°C with rotation. After three washes with lysis buffer, immunoblotting was performed with specific antibodies. Primary antibodies include anti-p53 (Santa Cruz Biotechnology, Cat# sc-126), anti-p-p53 Ser6, Ser9, Ser15, Ser20, Ser46, Ser392 (Cell Signaling Technology, Cat# 9285, 9288, 9286, 9287, 2521, 9281), anti-FLAG (Sigma, Cat# F7425), anti-p21 (Cell Signaling Technology, Cat# 2947), anti-β-actin (Abfrontier, Cat# LF-PA0207), and anti-VHH (Nanobody,Nb) (GenScript, Cat# A01861). Secondary antibodies include anti-Rabbit IgG-HRP linked (BioRad Laboratories, Cat# 1706515) and anti-Mouse IgG-HRP-linked (BioRad Laboratories, Cat# 1706516). Uncropped blot images are provided in supplementary information.

### RNA extraction and qRT-PCR

Total RNA was extracted using TRIzol Reagent (Invitrogen, Cat# 15596026). Reverse transcription was performed using High Capacity RNA-to-cDNA Kit (Applied biosystems, Cat# 4387406) following the manufacturer’s instructions. qRT-PCR was performed using Fast SYBR Green Master Mix (Applied Biosystems, Cat# 4385612). Forward and Reverse primer sequences were obtained from previously published studies and are provided in Supplementary Table S2. Data were normalized to GAPDH mRNA levels.

### Cell proliferation assay

HCT116 stable cell lines were seeded in 96-well plates in triplicate at a density 5×10^3^ cells/well in 100 μl of RPMI1640 medium containing 10% FBS and 1% Penicillin/streptomycin. The cells were treated with or without doxycycline at a concentration of 1 μg/ml, and 5-Fluorouracil or Oxaliplatin as indicated. Cell proliferation was evaluated using the Cell Counting Kit 8 (Dojindo, Cat# CK04-01) according to the manufacturer’s instructions. The absorbance for each well was measured at 450 nm using a microplate reader.

### Colony formation assay

HCT116 stable cell line were seeded in 6-well plates in duplicate at a density 2 ×102. Cells grown for 10 days and doxycycline was added twice a week. The remaining colonies were washed with PBS (Welgene, Cat# LB 001-02), stained with crystal violet (Sigma-Aldrich, Cat# C0775) and then counted.

### Invasion assay

HCT116 stable cell line were seeded 2×10^5^ cells. Cell invasion was evaluated using Matrigel invasion chamber (Corning, Cat# 354480) according to the manufacturer’s protocols. After 72 h, the remaining cells in the bottom of the Matrigel chamber were stained with Hematoxylin & Eosin and then counted.

### Cell cycle analysis

To determine cell cycle distribution, flow cytometry was performed after propidium iodide staining. Cells were trypsinized and washed with PBS and fixed with 70% ethanol. After washing with PBS, cells were incubated with Propidium iodide (Invitrogen, Cat# P3566) and RNase A and analyzed by BD FACSVerse flow cytometer (BD Bioscience). Data were analyzed using FlowJo software ver10.8.1.

### Xenograft experiments

Doxycycline-inducible stable HCT116 cells expressing p53Nb-KA (2 ×106 cells) were subcutaneously injected into the left flank of 6-week-old female BALB/c-nude mice (Orient Bio). Once the average tumor size reached about 50 mm^3^, the mice were randomly divided into two groups, with one group receiving a diet containing doxycycline until the end of the experiment. Tumor sizes were measured using a digital caliper three times a week. This study was reviewed and approved by the Institutional Animal Care and Use Committee (IACUC) of the Research Institute for Future Medicine (RIFM) at Samsung Medical Center (Approval Code: 20250114004). RIFM is an AAALAC International accredited facility and adheres to the Institute of Laboratory Animal Resources (ILAR) guide.

### Statistical analysis

Data were analyzed using two-tailed Student’s *t* test and are presented as mean ± SEM. Statistical significance was defined as *p < 0.05, **p < 0.01 and ***p < 0.001.

## Supplementary information

Supplementary Tables and Figures

Uncropped Western blot image

## Data Availability

All the data generated during this research were included in the article.
