# Supplementary material for: Regulation of cellular states via targeted phosphorylation of p53 using a nanobody-coupled kinase system
Source: Cell Death Discov. 2025 Nov 10;11:527. doi: 10.1038/s41420-025-02821-1 (PMC12603232; doi:10.1038/s41420-025-02821-1)
Supplement: Supplementary file 1 — Supplementary Tables and Figures [file 41420_2025_2821_MOESM1_ESM.pdf]

Supplementary Table S1.

Table S1. Sequence of construct used in this study.

	**Coding sequences of constructs**
FLAG-GFPNb-Kinase	ATGGATTACAAGGATGACGACGATAAGGGATCCGTCCAACTGGTGGAGTCTGGTGGCGCTTTGGTGCAGCCAGG<br>TGGCTCTCTGCGTTTGTCCTGTGCCGCTTCTGGCTTCCCAGTGAACCGCTATTCCATGCGCTGGTATCGCCAGGC<br>TCCAGGCAAAGAGCGTGAGTGGGTAGCCGGTATGTCCAGCGCGGGTGATCGTAGCTCCTATGAAGACTCCGTGA<br>AGGGCCGTTTCACCATCAGCCGTGACGATGCCCGTAACACGGTGTATCTGCAAATGAACAGCTTGAAACCTGAA<br>GATACGGCCGTGTATTACTGTAATGTGAACGTGGGCTTCGAGTATTGGGGCCAAGGCACCCAGGTCACCGTCTCC<br>AGCGATATCAGTGCTGCAGTGACTGCAGGGAAGCTGGCACGGGCACCGGCCGACCCTGGGAAAGCCGGGGTCC<br>CCGGAGTTGCAGCTCCCGGAGCTCCGGCGGCGGCTCCACCGGCGAAAGAGATCCCGGAGGTCCTAGTGGACCC<br>ACGCAGCCGGCGGCGCTATGTGCGGGGCCGCTTTTTGGGCAAGGGCGGCTTTGCCAAGTGCTTCGAGATCTCGG<br>ACGCGGACACCAAGGAGGTGTTCGCGGGCAAGATTGTGCCTAAGTCTCTGCTGCTCAAGCCGCACCAGAGGGAG<br>AAGATGTCCATGGAAATATCCATTCACCGCAGCCTCGCCCACCAGCACGTCGTAGGATTCCACGGCTTTTTCGA<br>GGACAACGACTTCGTGTTCGTGGTGTTGGAGCTCTGCCGCCGGAGGTCTCTCCTGGAGCTGCACAAGAGGAGGA<br>AAGCCCTGACTGAGCCTGAGGCCCGATACTACCTACGGCAAATTGTGCTTGGCTGCCAGTACCTGCACCGAAAC<br>CGAGTTATTCATCGAGACCTCAAGCTGGGCAACCTTTTCCTGAATGAAGATCTGGAGGTGAAAATAGGGGATTTT<br>GGACTGGCAACCAAAGTCGAATATGACGGGGAGAGGAAGAAGACCCTGTGTGGGACTCCTAATTACATAGCTCC<br>CGAGGTGCTGAGCAAGAAAGGGCACAGTTTCGAGGTGGATGTGTGGTCCATTGGGTGTATCATGTATACCTTGTT<br>AGTGGGCAAACCACCTTTTGAGACTTCTTGCCTAAAAGAGACCTACCTCCGGATCAAGAAGAATGAATACAGTA<br>TTCCCAAGCACATCAACCCCGTGGCCGCCTCCCTCATCCAGAAGATGCTTCAGACAGATCCCACTGCCCGCCCA<br>ACCATTAACGAGCTGCTTAATGACGAGTTCTTTACTTCTGGCTATATCCCTGCCCGTCTCCCCATCACCTGCCTG<br>ACCATTCCACCAAGGTTTTCGATTGCTCCCAGCAGCCTGGACCCCAGCAACCGGAAGCCCCTCACAGTCCTCAA<br>TAAAGGCTTGGAGAACCCCTAA
FLAG-GFPNb-KA	ATGGATTACAAGGATGACGACGATAAGGGATCCGTCCAACTGGTGGAGTCTGGTGGCGCTTTGGTGCAGCCAGG<br>TGGCTCTCTGCGTTTGTCCTGTGCCGCTTCTGGCTTCCCAGTGAACCGCTATTCCATGCGCTGGTATCGCCAGGC<br>TCCAGGCAAAGAGCGTGAGTGGGTAGCCGGTATGTCCAGCGCGGGTGATCGTAGCTCCTATGAAGACTCCGTGA<br>AGGGCCGTTTCACCATCAGCCGTGACGATGCCCGTAACACGGTGTATCTGCAAATGAACAGCTTGAAACCTGAA<br>GATACGGCCGTGTATTACTGTAATGTGAACGTGGGCTTCGAGTATTGGGGCCAAGGCACCCAGGTCACCGTCTCC<br>AGCGATATCAGTGCTGCAGTGACTGCAGGGAAGCTGGCACGGGCACCGGCCGACCCTGGGAAAGCCGGGGTCC<br>CCGGAGTTGCAGCTCCCGGAGCTCCGGCGGCGGCTCCACCGGCGAAAGAGATCCCGGAGGTCCTAGTGGACCC<br>ACGCAGCCGGCGGCGCTATGTGCGGGGCCGCTTTTTGGGCAAGGGCGGCTTTGCCAAGTGCTTCGAGATCTCGG<br>ACGCGGACACCAAGGAGGTGTTCGCGGGCAAGATTGTGCCTAAGTCTCTGCTGCTCAAGCCGCACCAGAGGGAG<br>AAGATGTCCATGGAAATATCCATTCACCGCAGCCTCGCCCACCAGCACGTCGTAGGATTCCACGGCTTTTTCGA<br>GGACAACGACTTCGTGTTCGTGGTGTTGGAGCTCTGCCGCCGGAGGGATCTCCTGGAGCTGCACAAGAGGAGGA<br>AAGCCCTGACTGAGCCTGAGGCCCGATACTACCTACGGCAAATTGTGCTTGGCTGCCAGTACCTGCACCGAAAC<br>CGAGTTATTCATCGAGACCTCAAGCTGGGCAACCTTTTCCTGAATGAAGATCTGGAGGTGAAAATAGGGGATTTT<br>GGACTGGCAACCAAAGTCGAATATGACGGGGAGAGGAAGAAGGACCTGTGTGGGACTCCTAATTACATAGCTCC<br>CGAGGTGCTGAGCAAGAAAGGGCACAGTTTCGAGGTGGATGTGTGGTCCATTGGGTGTATCATGTATACCTTGTT<br>AGTGGGCAAACCACCTTTTGAGACTTCTTGCCTAAAAGAGACCTACCTCCGGATCAAGAAGAATGAATACAGTA<br>TTCCCAAGCACATCAACCCCGTGGCCGCCTCCCTCATCCAGAAGATGCTTCAGACAGATCCCACTGCCCGCCCA<br>ACCATTAACGAGCTGCTTAATGACGAGTTCTTTACTTCTGGCTATATCCCTGCCCGTCTCCCCATCACCTGCCTG<br>ACCATTCCACCAAGGTTTTCGATTGCTCCCAGCAGCCTGGACCCCAGCAACCGGAAGCCCCTCACAGTCCTCAA<br>TAAAGGCTTGGAGAACCCCTAA
FLAG-GFPNb-KD	ATGGATTACAAGGATGACGACGATAAGGGATCCGTCCAACTGGTGGAGTCTGGTGGCGCTTTGGTGCAGCCAGG<br>TGGCTCTCTGCGTTTGTCCTGTGCCGCTTCTGGCTTCCCAGTGAACCGCTATTCCATGCGCTGGTATCGCCAGGC<br>TCCAGGCAAAGAGCGTGAGTGGGTAGCCGGTATGTCCAGCGCGGGTGATCGTAGCTCCTATGAAGACTCCGTGA<br>AGGGCCGTTTCACCATCAGCCGTGACGATGCCCGTAACACGGTGTATCTGCAAATGAACAGCTTGAAACCTGAA<br>GATACGGCCGTGTATTACTGTAATGTGAACGTGGGCTTCGAGTATTGGGGCCAAGGCACCCAGGTCACCGTCTCC<br>AGCGATATCAGTGCTGCAGTGACTGCAGGGAAGCTGGCACGGGCACCGGCCGACCCTGGGAAAGCCGGGGTCC<br>CCGGAGTTGCAGCTCCCGGAGCTCCGGCGGCGGCTCCACCGGCGAAAGAGATCCCGGAGGTCCTAGTGGACCC<br>ACGCAGCCGGCGGCGCTATGTGCGGGGCCGCTTTTTGGGCAAGGGCGGCTTTGCCAAGTGCTTCGAGATCTCGG<br>ACGCGGACACCAAGGAGGTGTTCGCGGGCAAGATTGTGCCTAAGTCTCTGCTGCTCAAGCCGCACCAGAGGGAG<br>AAGATGTCCATGGAAATATCCATTCACCGCAGCCTCGCCCACCAGCACGTCGTAGGATTCCACGGCTTTTTCGA<br>GGACAACGACTTCGTGTTCGTGGTGTTGGAGCTCTGCCGCCGGAGGTCTCTCCTGGAGCTGCACAAGAGGAGGA<br>AAGCCCTGACTGAGCCTGAGGCCCGATACTACCTACGGCAAATTGTGCTTGGCTGCCAGTACCTGCACCGAAAC<br>CGAGTTATTCATCGAAACCTCAAGCTGGGCAACCTTTTCCTGAATGAAGATCTGGAGGTGAAAATAGGGGATTTT<br>GGACTGGCAACCAAAGTCGAATATGACGGGGAGAGGAAGAAGACCCTGTGTGGGACTCCTAATTACATAGCTCC<br>CGAGGTGCTGAGCAAGAAAGGGCACAGTTTCGAGGTGGATGTGTGGTCCATTGGGTGTATCATGTATACCTTGTT<br>AGTGGGCAAACCACCTTTTGAGACTTCTTGCCTAAAAGAGACCTACCTCCGGATCAAGAAGAATGAATACAGTA<br>TTCCCAAGCACATCAACCCCGTGGCCGCCTCCCTCATCCAGAAGATGCTTCAGACAGATCCCACTGCCCGCCCA<br>ACCATTAACGAGCTGCTTAATGACGAGTTCTTTACTTCTGGCTATATCCCTGCCCGTCTCCCCATCACCTGCCTG<br>ACCATTCCACCAAGGTTTTCGATTGCTCCCAGCAGCCTGGACCCCAGCAACCGGAAGCCCCTCACAGTCCTCAA<br>TAAAGGCTTGGAGAACCCCTAA

FLAG-p53Nb-KA	ATGGATTACAAGGATGACGACGATAAGGGATCCATGGCCCAGGTGCAGCTGCAGGAGTCTGGGGGAGGATTGGT<br>GCAGGCTGGGGGCTCTCTGAGACTCTCCTGTGCAGCCTCTGAACGCACTTTCAGTACCTATGCCATGGGCTGGTT<br>CCGCCAGGCTCCAGGGAGGGAGCGTGAGTTTCTGGCACAGATTAACTGGAGTGGTACCACCACATACTATGCAG<br>AGTCCGTGAAGGACCGAACAACCATCTCCAGAGACAACGCCAAGAACACGGTGTATCTGGAAATGAACAACCT<br>GAACGCCGATGACACGGGCATCTATTTCTGTGCAGCACACCCTCAGCGGGGCTGGGGCTCCACTTTAGGTTGGA<br>CTTACTGGGGCCAGGGGACCCAGGTCACCGTCTCCTCGGCCAGCGCTAGCGGCGGAGGCGGATCTGGTACCAGT<br>GCTGCAGTGACTGCAGGGAAGCTGGCACGGGCACCGGCCGACCCTGGGAAAGCCGGGGTCCCCGGAGTTGCAG<br>CTCCCGGAGCTCCGGCGGCGGCTCCACCGGCGAAAGAGATCCCGGAGGTCCTAGTGGACCCACGCAGCCGGCG<br>GCGCTATGTGCGGGGCCGCTTTTTGGGCAAGGGCGGCTTTGCCAAGTGCTTCGAGATCTCGGACGCGGACACCA<br>AGGAGGTGTTCGCGGGCAAGATTGTGCCTAAGTCTCTGCTGCTCAAGCCGCACCAGAGGGAGAAGATGTCCATG<br>GAAATATCCATTCACCGCAGCCTCGCCCACCAGCACGTCGTAGGATTCCACGGCTTTTTCGAGGACAACGACTT<br>CGTGTTCGTGGTGTTGGAGCTCTGCCGCCGGAGGGATCTCCTGGAGCTGCACAAGAGGAGGAAAGCCCTGACTG<br>AGCCTGAGGCCCGATACTACCTACGGCAAATTGTGCTTGGCTGCCAGTACCTGCACCGAAACCGAGTTATTCAT<br>CGAGACCTCAAGCTGGGCAACCTTTTCCTGAATGAAGATCTGGAGGTGAAAATAGGGGATTTTGGACTGGCAAC<br>CAAAGTCGAATATGACGGGGAGAGGAAGAAGGACCTGTGTGGGACTCCTAATTACATAGCTCCCGAGGTGCTGA<br>GCAAGAAAGGGCACAGTTTCGAGGTGGATGTGTGGTCCATTGGGTGTATCATGTATACCTTGTTAGTGGGCAAAC<br>CACCTTTTGAGACTTCTTGCCTAAAAGAGACCTACCTCCGGATCAAGAAGAATGAATACAGTATTCCCAAGCAC<br>ATCAACCCCGTGGCCGCCTCCCTCATCCAGAAGATGCTTCAGACAGATCCCACTGCCCGCCCAACCATTAACGA<br>GCTGCTTAATGACGAGTTCTTTACTTCTGGCTATATCCCTGCCCGTCTCCCCATCACCTGCCTGACCATTCCACC<br>AAGGTTTTCGATTGCTCCCAGCAGCCTGGACCCCAGCAACCGGAAGCCCCTCACAGTCCTCAATAAAGGCTTGG<br>AGAACCCCTAA
FLAG-p53Nb-KD	ATGGATTACAAGGATGACGACGATAAGGGATCCATGGCCCAGGTGCAGCTGCAGGAGTCTGGGGGAGGATTGGT<br>GCAGGCTGGGGGCTCTCTGAGACTCTCCTGTGCAGCCTCTGAACGCACTTTCAGTACCTATGCCATGGGCTGGTT<br>CCGCCAGGCTCCAGGGAGGGAGCGTGAGTTTCTGGCACAGATTAACTGGAGTGGTACCACCACATACTATGCAG<br>AGTCCGTGAAGGACCGAACAACCATCTCCAGAGACAACGCCAAGAACACGGTGTATCTGGAAATGAACAACCT<br>GAACGCCGATGACACGGGCATCTATTTCTGTGCAGCACACCCTCAGCGGGGCTGGGGCTCCACTTTAGGTTGGA<br>CTTACTGGGGCCAGGGGACCCAGGTCACCGTCTCCTCGGCCAGCGCTAGCGGCGGAGGCGGATCTGGTACCAGT<br>GCTGCAGTGACTGCAGGGAAGCTGGCACGGGCACCGGCCGACCCTGGGAAAGCCGGGGTCCCCGGAGTTGCAG<br>CTCCCGGAGCTCCGGCGGCGGCTCCACCGGCGAAAGAGATCCCGGAGGTCCTAGTGGACCCACGCAGCCGGCG<br>GCGCTATGTGCGGGGCCGCTTTTTGGGCAAGGGCGGCTTTGCCAAGTGCTTCGAGATCTCGGACGCGGACACCA<br>AGGAGGTGTTCGCGGGCAAGATTGTGCCTAAGTCTCTGCTGCTCAAGCCGCACCAGAGGGAGAAGATGTCCATG<br>GAAATATCCATTCACCGCAGCCTCGCCCACCAGCACGTCGTAGGATTCCACGGCTTTTTCGAGGACAACGACTT<br>CGTGTTCGTGGTGTTGGAGCTCTGCCGCCGGAGGTCTCTCCTGGAGCTGCACAAGAGGAGGAAAGCCCTGACTG<br>AGCCTGAGGCCCGATACTACCTACGGCAAATTGTGCTTGGCTGCCAGTACCTGCACCGAAACCGAGTTATTCAT<br>CGAAACCTCAAGCTGGGCAACCTTTTCCTGAATGAAGATCTGGAGGTGAAAATAGGGGATTTTGGACTGGCAAC<br>CAAAGTCGAATATGACGGGGAGAGGAAGAAGACCCTGTGTGGGACTCCTAATTACATAGCTCCCGAGGTGCTGA<br>GCAAGAAAGGGCACAGTTTCGAGGTGGATGTGTGGTCCATTGGGTGTATCATGTATACCTTGTTAGTGGGCAAAC<br>CACCTTTTGAGACTTCTTGCCTAAAAGAGACCTACCTCCGGATCAAGAAGAATGAATACAGTATTCCCAAGCAC<br>ATCAACCCCGTGGCCGCCTCCCTCATCCAGAAGATGCTTCAGACAGATCCCACTGCCCGCCCAACCATTAACGA<br>GCTGCTTAATGACGAGTTCTTTACTTCTGGCTATATCCCTGCCCGTCTCCCCATCACCTGCCTGACCATTCCACC<br>AAGGTTTTCGATTGCTCCCAGCAGCCTGGACCCCAGCAACCGGAAGCCCCTCACAGTCCTCAATAAAGGCTTGG<br>AGAACCCCTAA

Supplementary Table S2.

Table S2. qPCR primer sets used in this study.

Gene		Sequence	Reference
p53	Fwd	GCCATCTACAAGCAGTCACAG	(1)
	Rev	TCATCCAAATACTCCACACGC	
p21	Fwd	TGTCACTGTCTTGTACCCTTG	(2)
	Rev	GGCGTTTGGAGTGGTAGAA	
MDM2	Fwd	CATTGAACCTTGTGTGATTTGTC	(3)
	Rev	GCAGGGCTTATTCCTTTTCTTTA	
PUMA	Fwd	ACGACCTCAACGCACAGTACG	(4)
	Rev	TCCCATGATGAGATTGTACAGGAC	
GADD45A	Fwd	ACTGCGTGCTGGTGACGAAT	(5)
	Rev	GTTGACTTAAGGCAGGATCCTTCCA	
NOXA	Fwd	GGAGATGCCTGGGAAGAAGG	(6)
	Rev	TCCTGAGCAGAAGAGTTTGG	
BIM	Fwd	ATGTCTGACTCTGACTCTCG	(6)
	Rev	CCTTGTGGCTCTGTCTGTAG	
BAX	Fwd	TTTGCTTCAGGGTTTCATCCA	(7)
	Rev	CTCCATGTTACTGTCCAGTTCGT	
GAPDH	Fwd	TGCACCACCAACTGCTTAGC	(8)
	Rev	GGCATGGACTGTGGTCATGAG	

Supplementary Fig. S1

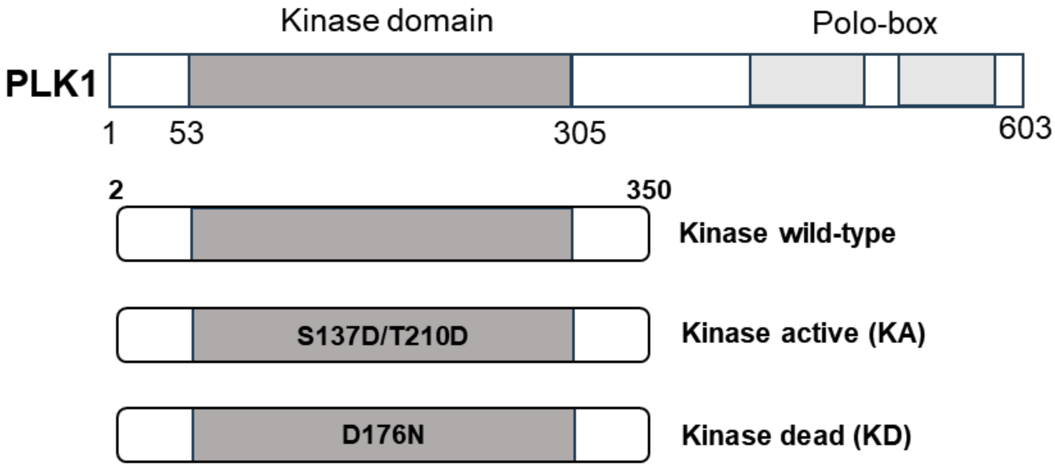

Fig. S1. Polo like kinase 1 (PLK1) kinase domain constructs.

Schematic representation of PLK1 kinase domain constructs used in the study. The kinase-active (KA) construct incorporates the mutations S137D and T210D within the kinase domain. The kinase-dead (KD) construct contains the mutation D176N within the kinase domain. These mutations were designed to create a fully functional kinase (KA), and a kinase-deficient version (KD). Amino acid positions are indicated.

Supplementary Fig. S2

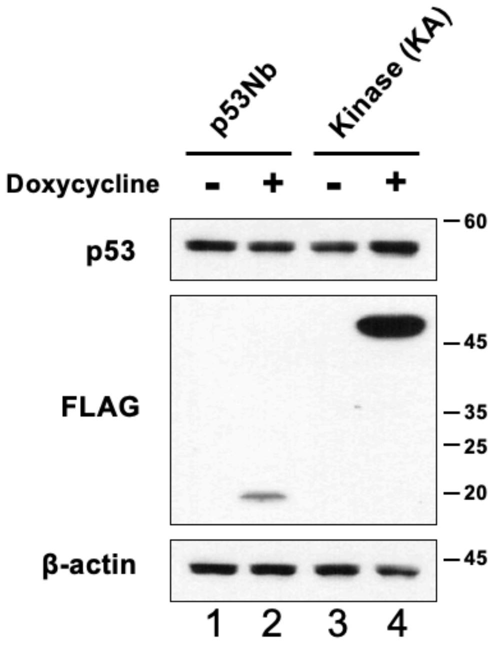

Fig. S2. Requirement of both nanobody and kinase domain for p53 stabilization.

Cells expressing only the p53 nanobody (lanes 1 and 2) or only the kinase domain (lanes 3 and 4) were treated with or without doxycycline. The lack of p53 upregulation in all lanes demonstrates that both components are required for p53 stabilization. β-actin is a loading control.

References

1. Xia W, Jiang P. p53 promotes antiviral innate immunity by driving hexosamine metabolism. Cell Rep. 2024;43(2):113724.
2. Langenberg T, Gallardo R, van der Kant R, Louros N, Michiels E, Duran-Romana R, et al. Thermodynamic and Evolutionary Coupling between the Native and Amyloid State of Globular Proteins. Cell Rep. 2020;31(2):107512.
3. Li M, Zhang X, Zhou WJ, Chen YH, Liu H, Liu L, et al. Hsp90 inhibitor BIIB021 enhances triptolide-induced apoptosis of human T-cell acute lymphoblastic leukemia cells in vitro mainly by disrupting p53-MDM2 balance. Acta Pharmacol Sin. 2013;34(12):1545-53.
4. Wang L, Zhu J, Fang M, Zhang T, Xie H, Wang N, et al. Inhibition of p53 deSUMOylation exacerbates puromycin aminonucleoside-induced apoptosis in podocytes. Int J Mol Sci. 2014;15(11):21314-30.
5. Lopez-Riego M, Plodowska M, Lis-Zajecka M, Jeziorska K, Tetela S, Wegierek-Ciuk A, et al. The DNA damage response to radiological imaging: from ROS and gammaH2AX foci induction to gene expression responses in vivo. Radiat Environ Biophys. 2023;62(3):371-93.
6. Delannoy A, Wilhelm E, Eilebrecht S, Alvarado-Cuevas EM, Benecke AG, Bell B. BIM and NOXA are mitochondrial effectors of TAF6delta-driven apoptosis. Cell Death Dis. 2018;9(2):70.
7. Seifaddinipour M, Farghadani R, Namvar F, Bin Mohamad J, Muhamad NA. In Vitro and In Vivo Anticancer Activity of the Most Cytotoxic Fraction of Pistachio Hull Extract in Breast Cancer. Molecules. 2020;25(8).
8. Jin R, Song G, Chai J, Gou X, Yuan G, Chen Z. Effects of concentrated growth factor on proliferation, migration, and differentiation of human dental pulp stem cells in vitro. J Tissue Eng. 2018;9:2041731418817505.
