# Supplementary material for: Regulation of cellular states via targeted phosphorylation of p53 using a nanobody-coupled kinase system
Source: Cell Death Discov. 2025 Nov 10;11:527. doi: 10.1038/s41420-025-02821-1 (PMC12603232; doi:10.1038/s41420-025-02821-1)

Uncropped blot image

Fig. 1

B

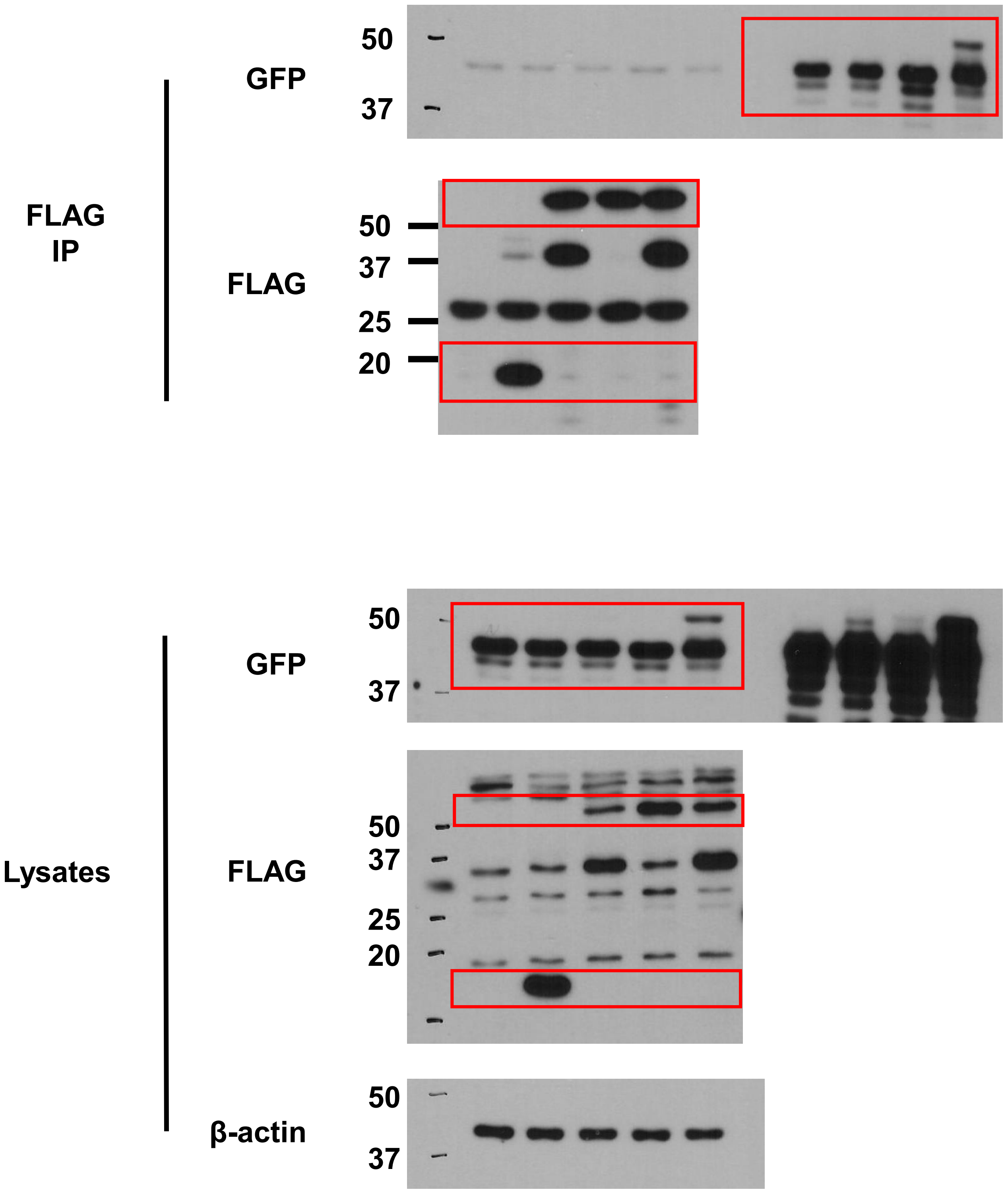

Fig. 1

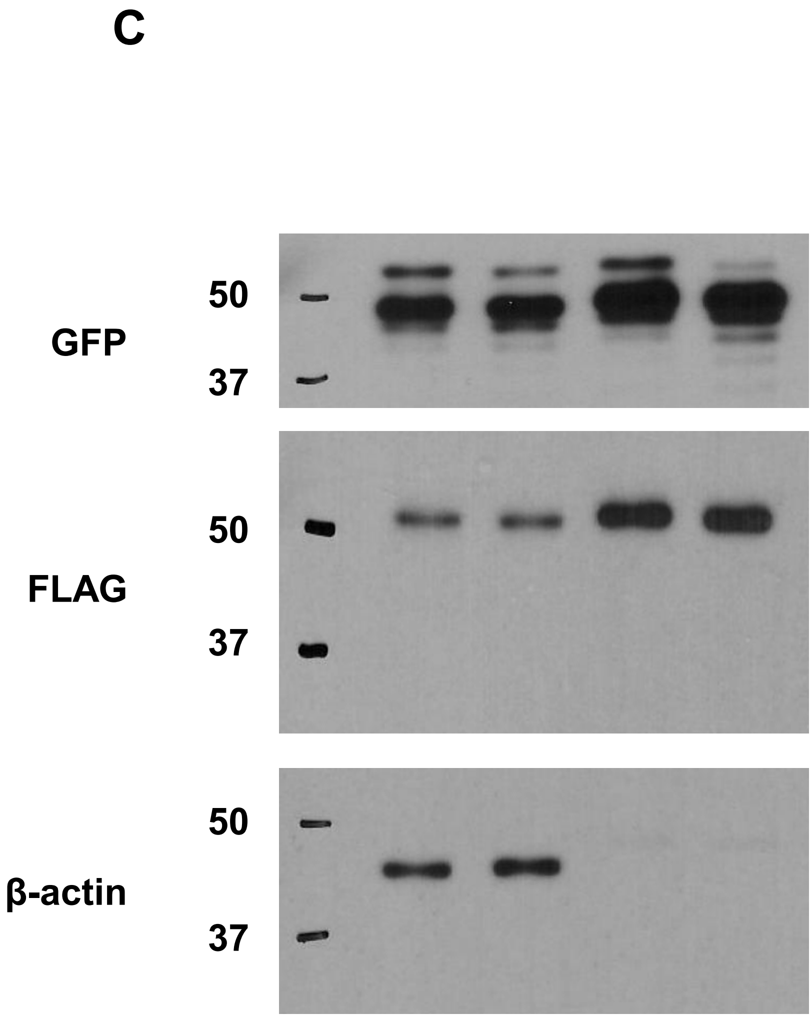

Fig. 2

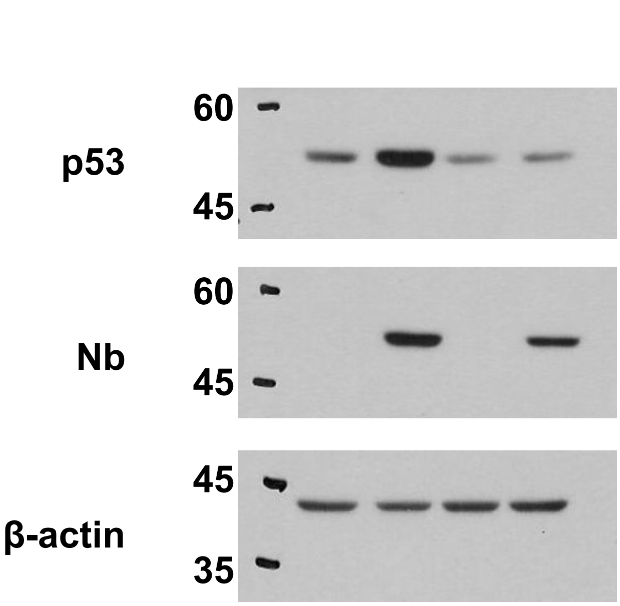

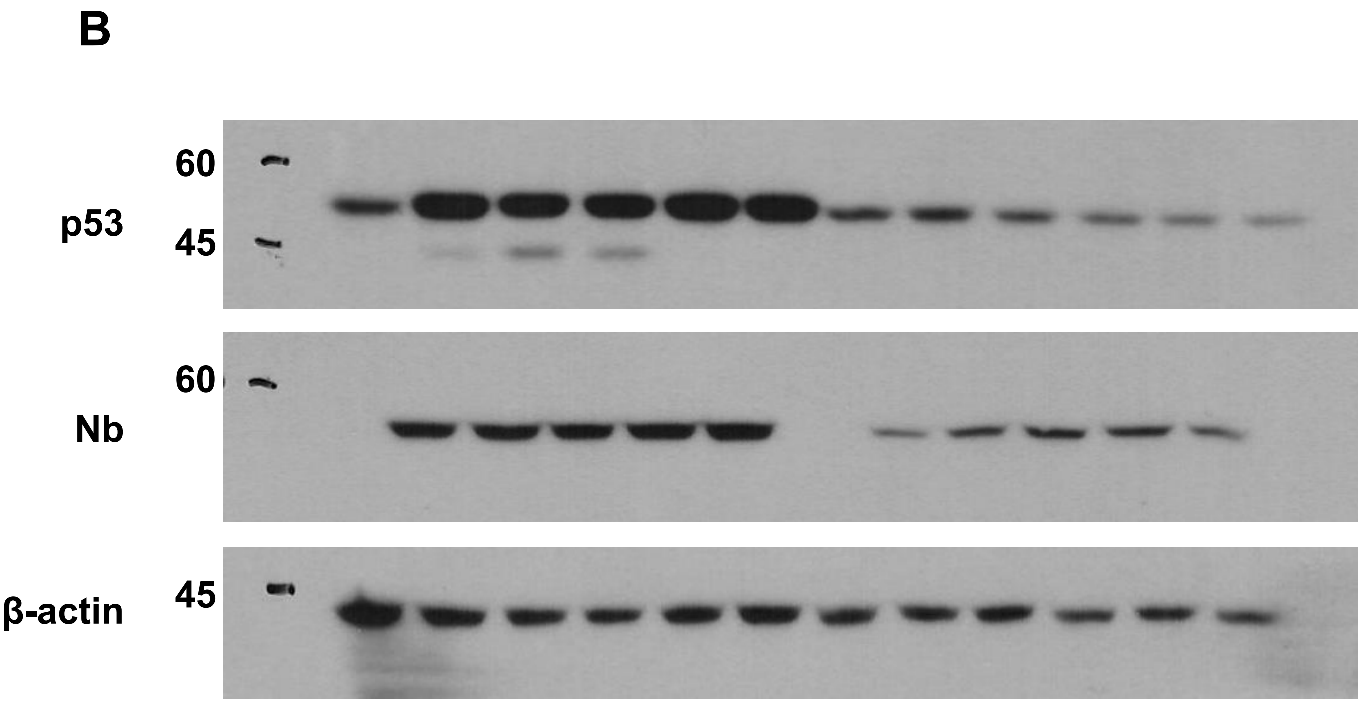

Fig. 2

C

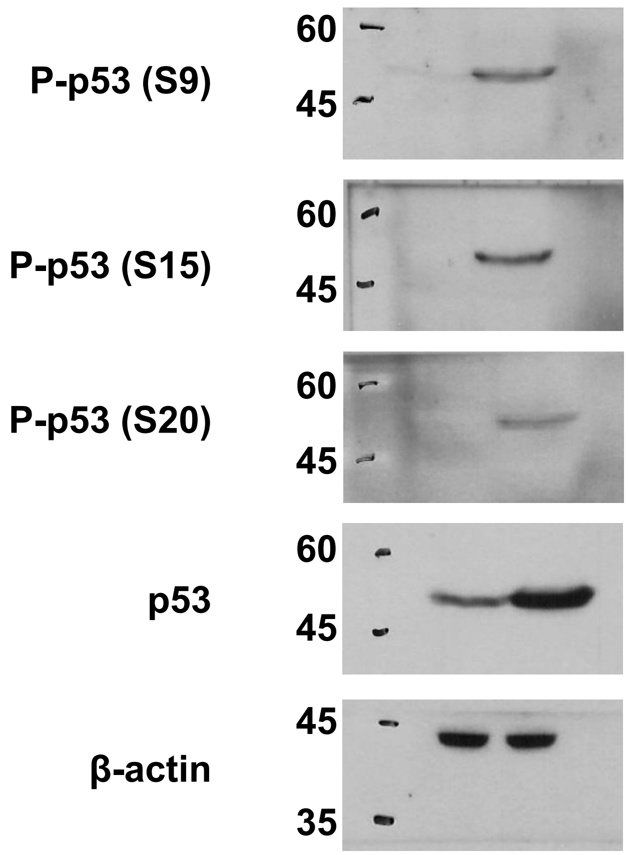

Fig. 2

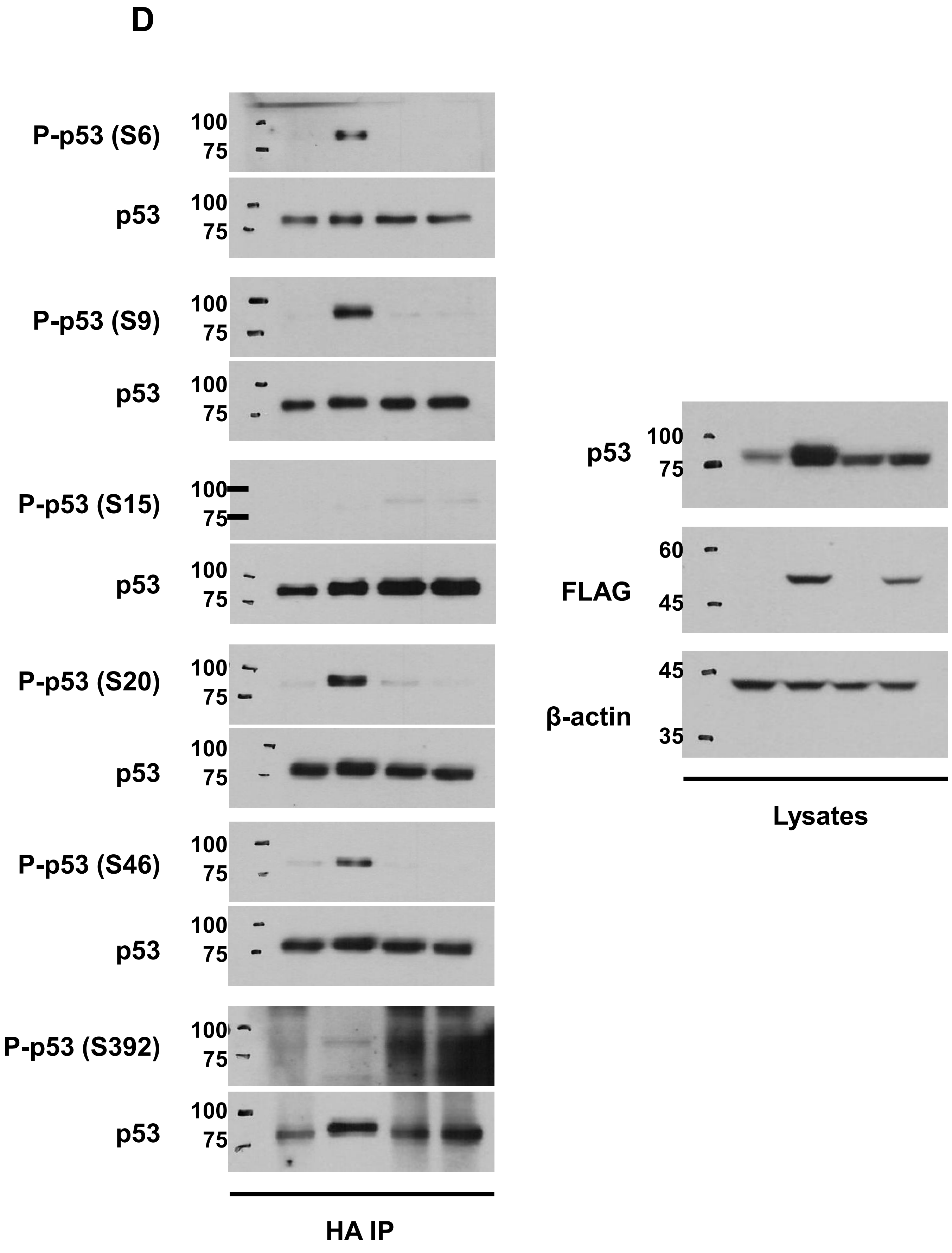
D
P-p53 (S6)
100
75
p53
100
75
P-p53 (S9)
100
75
p53
100
75
P-p53 (S15)
100
75
p53
100
75
P-p53 (S20)
100
75
p53
100
75
P-p53 (S46)
100
75
p53
100
75
P-p53 (S392)
100
75
p53
100
75
HA IP
p53
100
75
FLAG
60
45
β-actin
45
35
Lysates

Fig. 2

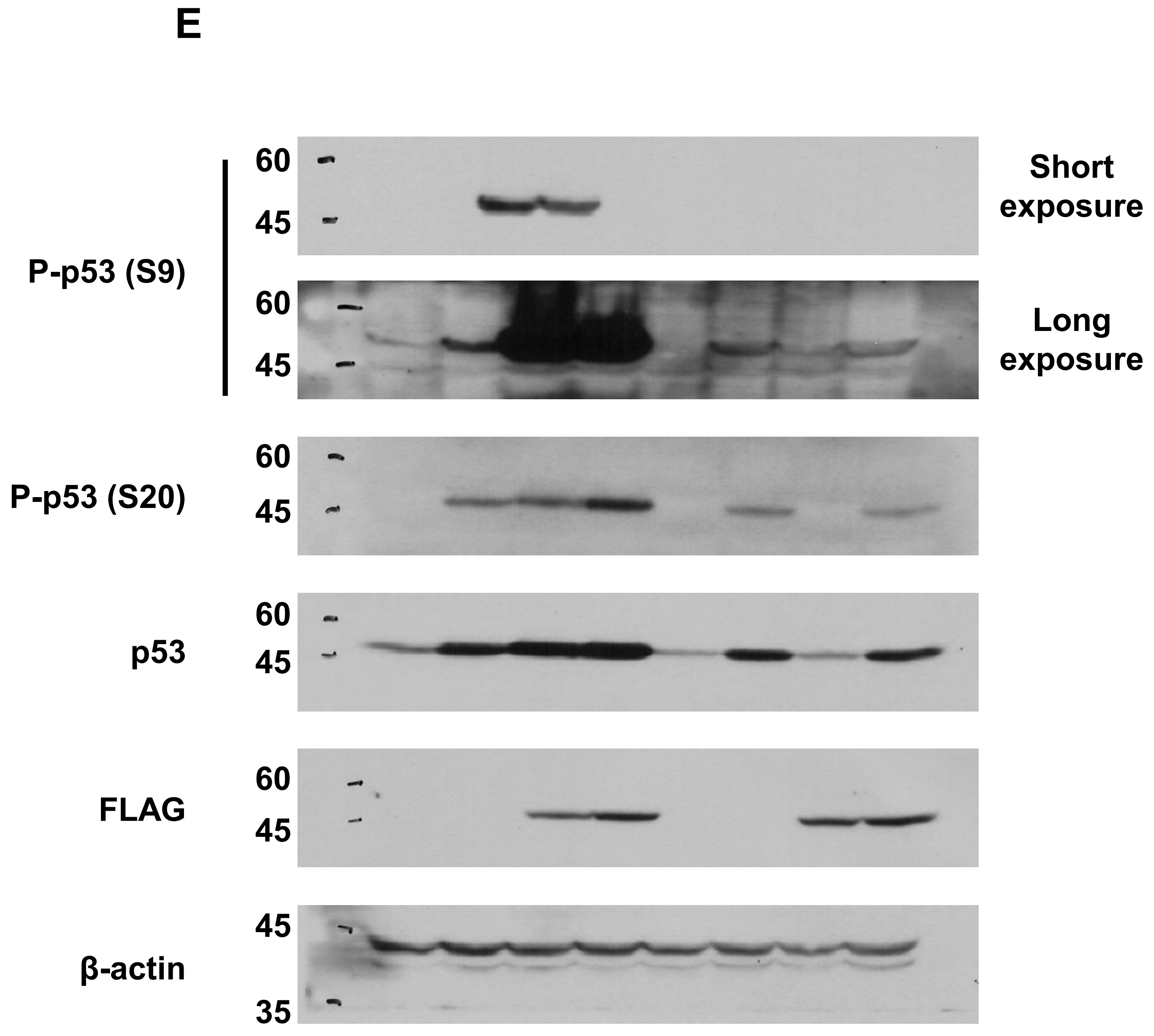
E
60
45
Short exposure
P-p53 (S9)
60
45
Long exposure
60
P-p53 (S20)
45
60
p53
45
60
FLAG
45
45
β-actin
35

Fig. 3

A

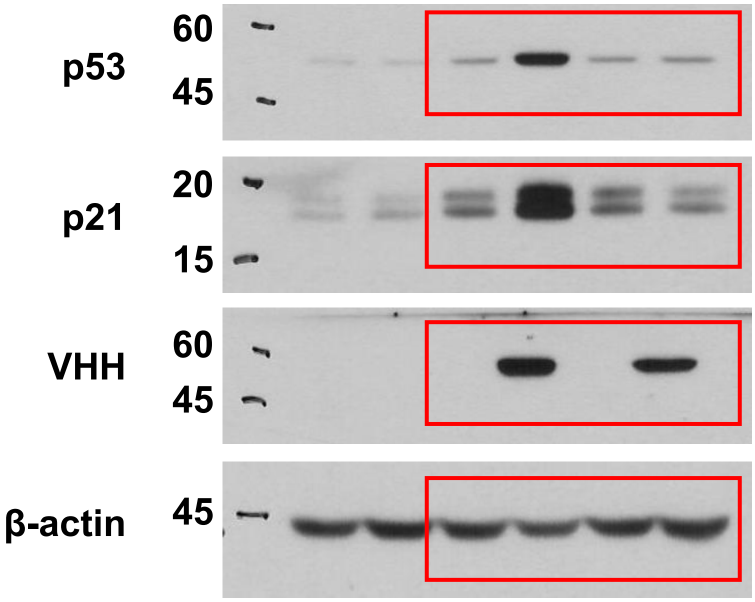

Supplementary Fig. S2

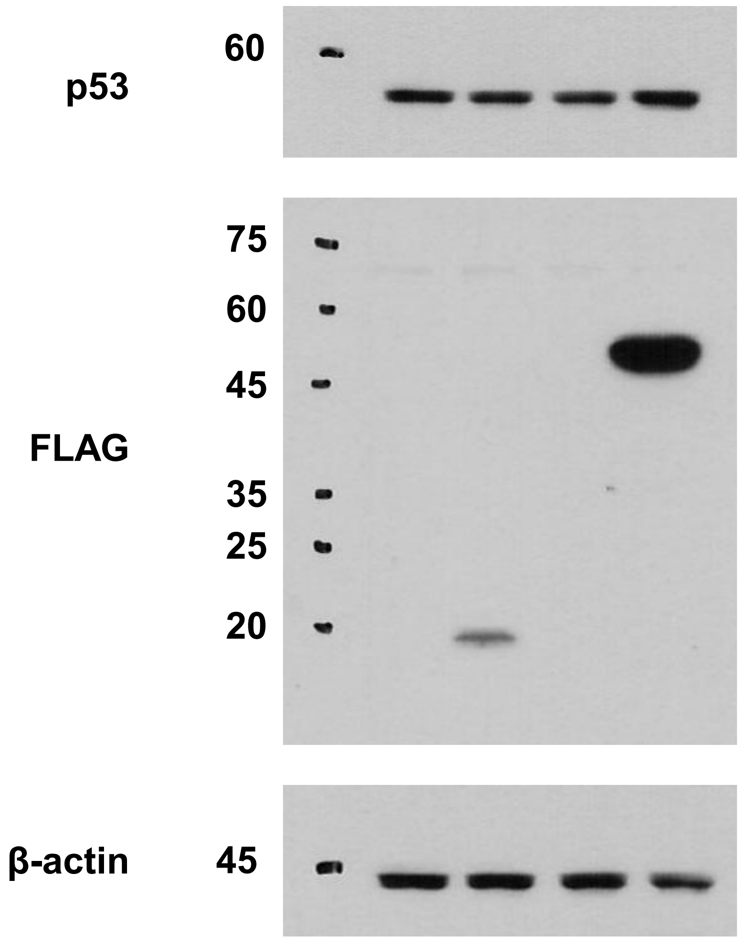

Supplement: Supplementary file 2 — Uncropped Western blot image [file 41420_2025_2821_MOESM2_ESM.pdf]
